# Emergent Roles of Circular RNAs in Metabolism and Metabolic Disorders

**DOI:** 10.3390/ijms23031032

**Published:** 2022-01-18

**Authors:** Yueh-Lin Wu, Hsiao-Fen Li, Hsi-Hsien Chen, Heng Lin

**Affiliations:** 1 Graduate Institute of Clinical Medicine, College of Medicine, Taipei Medical University, Taipei 110, Taiwan; vincewu168@gmail.com; 2 Division of Nephrology, Department of Internal Medicine, Wei-Gong Memorial Hospital, Miaoli 351, Taiwan; 3 Institute of Molecular and Genomic Medicine, College of Medicine, National Health Research Institutes, Miaoli 350, Taiwan; 4 TMU Research Center of Urology and Kidney, Taipei Medical University, Taipei 110, Taiwan; 5 Department of Physiology, School of Medicine, College of Medicine, Taipei Medical University, Taipei 110, Taiwan; bubble0728@gmail.com; 6 Division of Nephrology, Department of Internal Medicine, School of Medicine, College of Medicine, Taipei Medical University, Taipei 110, Taiwan; 7 Division of Nephrology, Department of Internal Medicine, Taipei Medical University Hospital, Taipei 110, Taiwan

**Keywords:** circRNA, obesity, diabetes, NAFLD, NASH

## Abstract

Circular RNAs (circRNAs) are an emerging group of long non-coding RNAs (lncRNAs) and have attracted attention again according to the progress in high-throughput sequencing in recent years. circRNAs are genome transcripts produced from pre-messenger (m)RNA regions in a specific process called “back-splicing,” which forms covalently closed continuous loops. Due to their lack of a 5’ cap and 3’ poly-adenylated tails, circRNAs are remarkably more stable than linear RNAs. Functionally, circRNAs can endogenously sponge to microRNAs, interact with RNA-binding proteins (RBPs), or translate themselves. Moreover, circRNAs can be expressed in cell type- or tissue-specific expression patterns. Therefore, they are proposed to play essential roles in fine-tuning our body’s homeostasis by regulating transcription and translation processes. Indeed, there has been accumulating emergent evidence showing that dysregulation of circRNAs can lead to metabolic disorders. This study explored the current knowledge of circRNAs that regulate molecular processes associated with glucose and lipid homeostasis and related pathogeneses of metabolic disorders. We also suggest the potential role of circRNAs as disease biomarkers and therapeutic targets.

## 1. Introduction

Statistics from the World Health Organization from 2000 to 2019 reveal that “non-communicable diseases” accounted for seven of the top 10 causes of death worldwide. Heart disease still ranks first among all diseases [1]. Diabetes is also among the top 10, and the related death toll increased during that period [1]. This phenomenon reflects changes in human lives in modern times. By analyzing risk factors of cardiovascular disease and diabetes, one can find many similarities, with the most notorious one being metabolic syndrome (MetS). MetS describes a clustering of the dysregulation of several metabolic processes, including hyperglycemia, hyperlipidemia, and abnormal adipose deposition. Per the concept of syndrome X, as proposed by Dr. Reaven, a pioneer in metabolic syndrome research, this clustering phenomenon is closely related to insulin resistance (IR) [2].

IR refers to the phenomenon of an insufficient response by fat cells, muscle cells, and liver cells to normal circulatory levels of insulin [3]. Several major mechanisms were suggested, including oxidative stress, inflammation, insulin receptor mutations, endoplasmic reticular (ER) stress, and mitochondrial dysfunction.

However, the exact underlying cause of IR has not been fully elucidated. Nevertheless, this phenomenon originates from interactions between genes and the environment, and this concept is still highly relevant.

Human genomic studies found that our genome is composed of about 3 million bases, but only 1%–2% form exons and are transcribed into proteins. In recent years, with advances in sequencing technologies, we have begun to understand that the transcription of noncoding regions is affected by environmental factors and thus changes in the performance of genes, especially in metabolic diseases, such as obesity and diabetes [4]. While infrastructural types, including ribosomal (r)RNAs, transfer (t)RNAs, and small nuclear (sn)RNAs, are typically constitutively expressed, regulatory types, such as micro (mi)RNAs, long noncoding (lnc)RNAs, circular (circ)RNAs, and piwi-interacting (pi)RNAs, were proposed to fine-tune target gene expressions [5]. These special ncRNAs are not necessarily transcribed into proteins but orchestrate gene regulatory networks in a hidden layer [5,6,7].

Of note among ncRNAs, circRNAs do not have a traditional linear structure but consist of a loop formed by covalent bonding (reverse splicing), and they lack a 5‘ cap and 3‘ poly-adenylated tails [8]. As a result of these features, circRNAs are not cut by exonucleases or RNases and are much more stable than most linear RNAs in cells. Therefore, it is reasonable to hypothesize that circRNAs might be involved in metabolic homeostasis, which was recently proven by emerging data. Hence, we discuss the relevant literature on relationships between circRNAs and metabolic dysregulation and highlight their roles as biomarkers and therapeutic targets in metabolism-related diseases.

## 2. The Biogenesis, Biology, and Characterization of circRNAs

Viroids, which are small, circular, single-stranded RNA molecules (of 246–401 nucleotides) that are uncoated and pathogenic to higher plants, were the first circRNA molecules to be discovered in 1976 [9]. A few years later, circRNAs were observed in the cytoplasmic fractions of eukaryotic cell lines by electron microscopy. However, due to a lack of suitable research tools, circRNAs were once considered to be “junk” products produced by aberrant RNA splicing. They have low abundances in cells and low sequence conservation, and received little attention until 1993, when it was discovered that Sry, the sex-determining gene, undergoes circular transcription in adult mouse testicles [10].

Recently, with advances in high-throughput sequencing (Seq) technologies, Salzman reported ~80 circular RNAs for the first time through the RNA-Seq method [11]. Since then, large numbers of circRNA molecules have been discovered. Jeck et al. detected more than 25,000 circRNAs in human fibroblasts; Memczak et al. identified 1950 human circRNAs and 1903 mouse species through RNA-Seq data combined with a human leukocyte database (81 of which were the same as human circRNAs) and 724 nematode circRNAs [12,13]. Of note, with these new discoveries, people began to recognize that circRNAs are abundant, diverse, and conserved molecules in tissue- and developmental stage-specific manners and might play critical roles in human biological processes [11,12,13].

### 2.1. The Biogenesis of circRNAs

**Figure 1:**
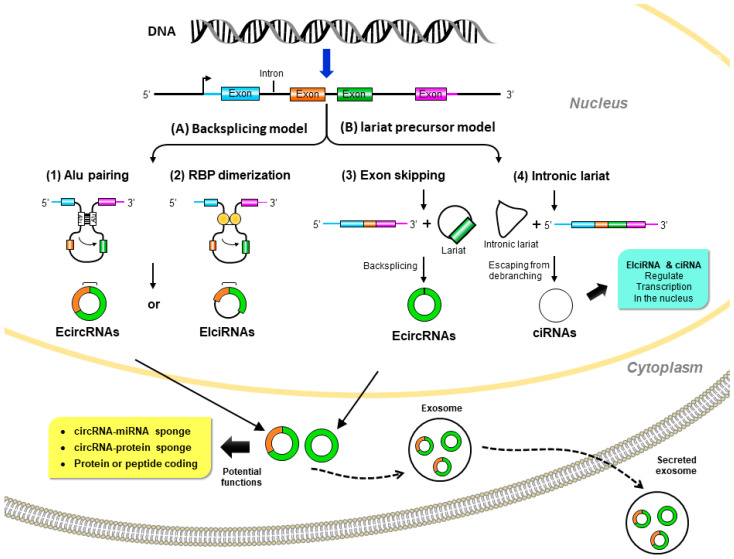
Biogenesis and potential biological functions of circular (circ)RNAs. (**A**) Back-splicing model: pre-mRNA is spliced in a non-canonical manner of “back-splicing” by (**1**) inverted repeat elements in long flanking intron pairs, such as Alu elements, or (**2**) dimerization of RNA-binding proteins (RBPs). During back-splicing, an upstream branch point attacks a downstream splice donor site to form exonic circRNAs or exon–intron circRNAs. (**B**) Lariat precursor model: pre-mRNA undergoes canonical splicing to generate a linear mRNA and a lariat precursor. (**3**) The lariat precursors with exon components might be generated from exon-skipping events and then further back-spliced to exonic circRNAs. Alternatively, (**4**) the intronic lariat precursors escape from the debranching step of canonical linear splicing to form intronic circRNAs. Exonic circRNAs are transported from the nucleus to the cytoplasm to function as miRNA sponges to inhibit miRNA activity; protein sponges (such as RBPs) affect protein functions and translocation or protein-coding to further translation. Exon–intron circRNAs and intronic circRNAs can interact with transcription complexes to regulate transcription in the nucleus.

Although it was proposed that circRNAs are derived from precursor messenger (m)RNAs (pre-mRNAs) by back-splicing, the mechanism of circRNA biogenesis still remains elusive. circRNAs are generally generated by joining an upstream 3′ splice site to a downstream 5′ splice site to form a covalently closed loop [14]. Based on the original sequences, circRNAs can mainly be classified in three categories: exonic circular (ecirc)RNAs [12,13,15], circular intronic (ci)RNAs [16], and exon–intron circular (EIci)RNAs [17]. The majority (over 80%) of circRNAs mainly reside in the cytoplasm, while ciRNAs and EIciRNAs are mainly found in nuclei [12,13,15]. Briefly, the formation of circRNAs can be classified into two popular models (Figure 1), the direct back-splicing model and the lariat precursor model.

#### 2.1.1. Direct Back-Splicing Model

In this model, paired elements flanking the downstream and upstream splices bring these sites into proximity to form a loop structure that favors back-splicing. Intron pairing, such as Alu elements [18], or the dimerization of RNA-binding proteins (RBPs), such as Muscleblind (MBL) [19] and Quaking (QKI) [20], initiate circularization in circRNA formation.

#### 2.1.2. Lariat Precursor Model

In addition to the back-splicing mechanism, circRNAs can also be produced from the processing of lariat sequences. Standard linear splicing converts an intron into a looped structure called a ‘lariat’ (like a lasso) [21]. The splicing machinery then generally eliminates the lariat. This mode includes the following two ways: (a) exon-skipping: during alternative linear splicing, a giant lariat containing the skipped exon can originate. These exon-containing lariats may further form mature circRNAs by back-splicing [22,23]. (b) Intron lariat: pre-mRNAs occur due to canonical linear splicing to from linear mRNA and a long intron lariat. Then the intron lariat that escaped from debranching is further back-spliced to produce mature circRNA [16].

Although the production of circRNA is still a mystery, the production efficiency is very low due to the sterically unfavorable connections between the downstream 5’ splice site and the upstream 3‘ splice site [24]. Nevertheless, because they are resistant to RNA exonucleases or RNase R, circRNAs can accumulate at higher concentration than linear RNAs in quiescent and post-mitotic cells, such as neurons [25]. At the same time, circRNAs are also rich in exosomes and could be found in extracellular fluid (like blood) at levels higher than linear RNA [26]. Therefore, circRNAs are considered good biomarkers for disease diagnosis.

### 2.2. Functions of circRNAs

The main functions of circRNAs are still not well understood, but growing data suggest that they play lots of essential roles in many biological processes. People proposed that circRNAs in different locations have diverse functions. While cytoplasmic circRNAs sponge miRNAs or proteins, nuclear circRNAs regulate transcription and alternative splicing events.

#### 2.2.1. circRNAs Act as miRNA Sponges

In 2013, two research teams published the first observations that circRNAs can indirectly modulate gene expressions at the posttranscriptional level by sequestering miRNA and mRNA interactions [11,12]. Those studies also noted that ciRS-7 circRNA contained more than 70 conserved binding sites for the miR-7 sponge, thereby regulating expression of miR-7 target mRNAs. Interestingly, ciRS-7-knockout mice had lower miR-7 levels, impaired sensorimotor gating, and enhanced miR-7 target genes, such as *Fos*, an immediate early gene [27]. That in vivo model suggested that interactions between ciRS-7 and miRNAs are important for essential brain functions.

Of note, ciRS-7’s inhibiting or protecting miR-7 from degradation may vary with the cellular context [27,28,29,30]. Finally, many other circRNAs, including circHIPK3 [31] and circBIRC6 [32], were shown to have miRNA sponging abilities. However, unlike ciRS-7, these circRNAs contain fewer miRNA binding sites than expected by chance [33].

#### 2.2.2. circRNAs Interact with Proteins

circRNAs can serve as protein decoys to regulate the function of RBPs, for example, circRNA poly(A)-binding protein nuclear 1 (circPABPN1) and circular antisense noncoding RNA in the INK4 locus (circANRIL). CircPABPN1 suppresses the translation of nuclear poly(A)-binding protein 1 (PABPN1) mRNA by sequestering the protein RBP Hu-antigen R (HUR) [34]. In vascular smooth muscle cells and macrophages, circANRIL sequesters pescadillo homologue 1 (PES1), an essential 60S preribosomal assembly factor, to impair rRNA maturation, resulting in atherosclerosis-related nucleolar stress, p53 activation, and cell apoptosis [35]. circRNAs can also function as protein scaffolds. circFoxo3 combined with the cell cycle proteins, cyclin-dependent kinase 2 (CDK2) and cyclin-dependent kinase inhibitor 1 (p21), form the circFoxo3–p21–CDK2 ternary complex and inhibit CDK2 function to repress cell cycle progression [36]. Recently, extensive screening of circRNA-RBP interactions was performed, and the concept of a circRNA-RBP interactome raises the emergent crucial regulatory roles in tumorigenesis and other vital cellular functions [37].

#### 2.2.3. circRNAs as Regulators of Transcription and Splicing

Recently, it was revealed that in the nucleus, both ciRNAs and EIciRNAs can modulate their parental gene transcription by interacting with upstream promoters, RNA polymerase II (Pol II), and other transcription machinery proteins [16,17,38]. Two ciRNAs, ci-ANKRD52 and ci-SIRT-7, enhance their parental gene expressions of ankyrin repeat domain 52 (ANKRD52) and sirtuin 7 (RIRT7), respectively [16,38]. By contrast, EIciRNAs, such as circEIF3J and circPAIP2, interact with the U1 small nuclear ribonucleoprotein (U1 snRNP) and RNA polymerase II (Pol II) via U1 snRNA-binding sites, thereby enhancing expressions of their parent genes [17].

Certain circRNAs also act on gene expressions trans-functionally by competing with linear splicing. For example, circMbl was proposed to function as a protein sponge, and it originates from the gene that encodes the splicing factor, muscleblind (mbl), in *Drosophila melanogaster* and the homologous gene, muscleblind-like protein 1 (MBNL1), in humans [17]. Interestingly, mbl itself directly regulates the biogenesis of circMbl by the presence of specific mbl-binding sites in the introns flanking the circularizable exon (also shown in MBNL) [17]. Therefore, this autoregulatory circuit may result in excess mbl or MBNL1 decreasing the production of its mRNA by promoting circMbl biogenesis, and the circMbl helps the linear splicing of the gene by tethering mbl or MBNL1 [17].

#### 2.2.4. circRNAs Translate to Proteins

Although circRNAs do not contain 5‘ caps or poly(A) tails, it was shown that circRNAs are translated in a cap-independent manner by the internal ribosome entry site (IRES) [39] and N6-methyl-adenosines (m6A) [40,41]. circ-ZNF609 was the first to be identified as a circRNA that makes a protein function involved in muscle development [42]. Additionally, the circular form of the long intergenic non-protein-coding RNA p53-induced transcript (LINC-PINT) can be translated into a small peptide involved in glioblastoma tumorigenesis [43]. Although there are many examples in the literature of circRNAs being translated into proteins or peptides, the functions of those molecules remain unknown.

## 3. circRNAs Regulate Insulin Signaling and β-Cell Function

**Figure 2:**
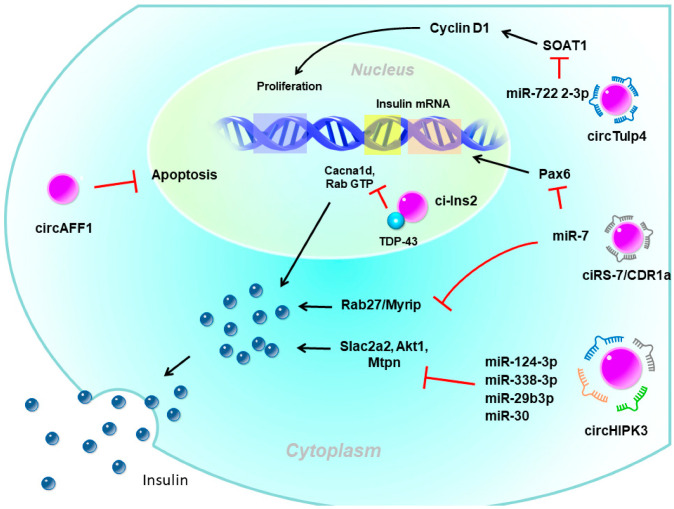
The potential role of circular (circ)RNAs in pancreatic β-cells. circHIPK3 and ciRS-7/CDR1a are exonic circRNAs mainly localized in the cytoplasm which act as miRNA sponges to enhance insulin secretion. ciRS-7/CDR1a also enhances insulin expression by the mi7 sponge. circTulp4 is also an exonic circRNA that sequesters miR-7222-3p to promote β-cell proliferation. circAFF1 is an exonic circRNA that inhibits β-cell apoptosis through an unknown mechanism. ci-Ins2 is an intronic circRNA mainly localized in the nucleus that interacts with the RNA-binding protein, TDP-43. The interaction between ci-Ins2 and TDP-43 promotes expression of the insulin secretory machinery. Pax6, paired box 6; SLc2a2, solute carrier family 2 member 2; Akt1, AKT serine/threonine kinase 1; Mtpn, myotrophin; Myrip, myosin VIIA and Rab interacting protein; Cacna1d, calcium voltage-gated channel subunit alpha1 D; TDP-43, TAR DNA-binding protein 43.

Pancreatic β-cells are the only source of insulin in the body and play a major role in glucose homeostasis. Type 1 diabetes, which mostly occurs in adolescents and children, is mainly caused by autoimmune problems, destroying pancreatic β-cells, leading to a lack of insulin and requiring insulin injections. In contrast, the main reason for type 2 diabetes (T2D) is IR due to dysfunction in insulin signaling and subsequent hyperglycemic status. The pancreas secretes more insulin to allow blood sugar to enter cells but the compensatory increase in insulin is limited. Over time, prolonged hyperglycemia leads to β-cell dysfunction, proliferation impairment, and apoptosis activation, forming a vicious circle of β-cell exhaustion. There is emerging evidence that circRNAs are involved in regulating β-cell functions and in diabetes development (Figure 2) [44,45,46,47].

### 3.1. circRNAs Regulate Insulin Secretion and β-Cell Function

circRNAs have been proposed to be involved in the regulation of insulin secretion. The most well-known circRNAs with such functions were ciRS-7/CDR1a and circHIPK3. CDR1as, which originates from the antisense transcript of the cerebellar degeneration-related protein 1 gene, was the first circRNA studied in pancreatic β-cells [48,49]. Since it contains more than 70 binding sites for miR-7, it is also named ciRS-7 (circRNA sponge for miR-7) [48,49]. Studies showed that miR-7 is one of the most common miRNAs expressed by β-cells and is a negative regulator of glucose-stimulated insulin secretion (GSIS) in β-cells [50]. Interestingly, while the overexpression of miR-7 in transgenic mouse β-cells causes diabetes due to impaired insulin secretion [50], overexpression of ciRS-7 leads to increased insulin secretion by islet cells by upregulating miR-7 target gene expressions, such as Myrip (myosin VIIA and Rab-interacting protein) [49]. ciRS-7 also enhances insulin transcription by regulating Pax6 (paired box 6) expression after sponging for miR-7 [49].

Furthermore, circHIPK3 is another very abundant islet circRNA and was reported to be reduced in islets of db/db mice [45]. Silencing of circHIPK3 caused defective insulin secretion, increased apoptosis, and reduced proliferation in MIN6B1 cells [45]. Furthermore, expressions of critical insulin secretion genes, such as Slc2a2, Akt1, and Mtpn, were downregulated upon circHIPK3 silencing [45]. Mechanistic studies proposed that these effects of circHIPK3 may be mediated via miRNA sponging, such as miR-124-3p, miR-338-3p, miR-29b-3p, and miR-30, which have known functions in β-cells [45]. Nevertheless, circHIPK3 silencing resulted in a decrease in the activity of a luciferase construct containing the 3‘ untranslated region (UTR) of human MTPN, which is known to be controlled by miR-124-3p [45].

Recently, ci-Ins2, derived from intron 2 of preproinsulin 2 (Ins2), also was suggested to be involved in insulin secretion. Silencing rat ci-Ins2 or ci-INS (the human homolog of ci-Ins2) was associated with reduced mRNA expressions that encode vital components of the insulin secretory machinery, including the voltage-dependent Ca2+ channel subunit, Cacna1d, and different targets and regulators of Rab3 GTPases [51]. TAR DNA-binding protein 43 (TDP-43)-knockout and ci-Ins2 silencing shared a common set of downregulated genes, especially genes involved in insulin exocytosis [52]. Interestingly, the level of ci-Ins2 remained unchanged in normoglycemic ob/ob mice but was reduced in islets of hyperglycemic db/db mice, as observed for circHIPK3 and ciRS-7 [45,51]. In addition, the level of human ci-INS was lower in islets of T2D donors and was inversely correlated with glycated hemoglobin (HbA1c) levels [51]. Taken together, these data suggest that ci-Ins2 might regulate insulin secretion in part through TDP-43 binding and contribute to β-cell dysfunction in the pathogenesis of T2D.

In addition to involving insulin secretion, circRNAs also impact β-cell apoptosis or proliferation. circAFF1 is exonic from the AF4/FMR2 family member 1 (AFF1) gene. The level of circAFF1 did not change in the type 1 or T2D model, but silencing circAFF1 enhanced β-cell apoptosis in vitro. Nevertheless, circAFF1 did not affect the capacities of proliferation, insulin content, or secretion in β-cells [45]. In contrast, circTulp4 promoted β-cell function by inducing the expression of the cholesterol esterification-related gene, sterol O-acyltransferase 1 (SOAT1), through inhibiting miR-7222-3p [53]. The accumulation of soat1 activated cyclin D1 expression and thus promoted cell cycle progression. Those findings suggested that circTulp4 might upregulate β-cell proliferation by the miR-7222-3p/soat1/cyclin D1 pathway [53].

Of note, more than 2600 circRNAs were recently identified in human pancreatic islets by CircleSeq [54]. One of the most abundant circRNAs in human islets, circCIRBP, demonstrated an association with the insulin secretory index in isolated human islets [54]. In addition, some islet circRNAs are expressed in peripheral blood, and circCAMSAP1 was correlated with T2D status [54].

### 3.2. circRNAs in IR

It is widely believed that IR is strongly associated with obesity and T2DM. Studies have proven the roles of miRNAs in regulating obesity and IR [55,56]. Until now, few comprehensive studies have specifically utilized the role of circRNAs in obesity-IR-T2DM settings. Nevertheless, a recent report of the involvement of circHIPK3 in hyperglycemia and IR by Cai et al. might open up this new world [57]. They demonstrated that increased circHIPK3 could sponge miR-192-5p, which targets and degrades Forkhead box protein O1 (*FOXO1*) mRNA, decreasing its expression in HepG2 cells [57]. *FOXO1* is a transcription factor that plays vital roles in regulating gluconeogenesis and glycogenolysis by insulin signaling [58,59]. Moreover, *FOXO1* inhibition increases uncoupling protein 1 (Ucp1) expression, which subsequently increases thermogenesis and reduces fat mass [60,61]. These findings suggest that circHIPK3 may increase IR and hyperglycemia by upregulating *FOXO1*. Even though those current studies did not directly link potential circRNA signatures and obesity-related IR, they highlight a future direction for further elucidation.

## 4. circRNAs in Adipocytes and Obesity

Obesity refers to a physical condition in which excess body fat accumulates and harms health. The standard of obesity is often measured by the body mass index (BMI), which is the weight (in kg) divided by the height (in m) squared. The waist–hip ratio is also a standard defined as obesity in men of greater than 0.9 m or women of greater than 0.85 m. In summary, we can understand that obesity is a state of excessive accumulation of adipose tissues and their uneven distribution. Therefore, exploring adipogenesis and lipid metabolism has become a critical basis for preventing obesity. Emergent data suggest that circRNAs orchestrate various aspects of lipid metabolism, such as adipogenesis, lipogenesis, and browning, in adipose tissues (Table 1).

### 4.1. circRNAs Regulate Adipose Tissue Formation

Pigs are similar to humans in anatomy and physiology, and, in particular, the brown fat of both disappears in adulthood. Therefore, pigs have become an excellent animal model for studying lipid metabolic diseases. Based on this, Li and colleagues compared the expression of circRNA in subcutaneous fat tissues of Large White pigs and Laiwu pigs. They found that in subcutaneous adipose tissues of Laiwu pigs, circRNA_26852 was significantly upregulated, and circRNA_11897 was significantly downregulated, and its target genes were involved in pathways related to adipocyte differentiation and lipid metabolism [62]. Based on similar ideas, Jiang et al. analyzed the circRNA expression profile of newborn calves and adult adipose tissues in Qinchuan cattle, a popular livestock breed with high muscle fat contents [63]. Results showed that circFUT10 was upregulated in adult fat [63]. Further molecular studies showed that circFUT10 could sponge let-7c to reduce its inhibition of PPARGC1B (peroxisome proliferator-activated receptor γ coactivator 1-β) and further regulate the proliferation and differentiation of adipocytes [63]. Similarly, Sun et al. compared circRNA expression profiles in human visceral preadipocytes and differentiated visceral adipocytes [64]. In total, 2215 significantly upregulated and 1865 significantly downregulated circRNAs were identified [64].

In addition to being involved in adipogenesis, circRNAs were also recently reported to be involved in the browning of white fat. Zhang et al. found that the level of plasma exosomal ciRS-133 was assuredly correlated with brown adipose tissues in gastric cancer patients [65]. CiRS-133, delivered by exosomes, promotes the browning of preadipocytes by absorbing miR-133 and upregulating PRDM16 (PR domain containing 16) [65]. In addition, the circ005661 sequence called circNrxn2, transcribed from the *Nrxn2* gene, has a potential binding site for miR-103, which is abundant in adipose tissues and regulates browning [66]. Overexpression of circNrxn2 improved white adipose tissue browning in high-fat diet (HFD)-fed mice [66]. Results of co-culture of macrophages and adipocytes indicated that circNrxn2 promoted white adipose tissue browning by increasing the polarization of M2 macrophages [66].

**Table 1:** Current roles of circular (circ)RNAs in adipocyte lipid metabolism and obesity.

circRNA	Expression Pattern in Disease	miRNA/ Protein	Effector Target	Biological Function	Cell/Tissue Type	Molecular Assay	References
circRNA_11897	Down	miR-27b-3p	SCD	Lipogenesis↑	Large White and Laiwu pigs adipose tissue (SC)	-	[60]
circRNA_26852	Up	miR-486	ABHD5	Lipolysis↑	Large White and Laiwu pigs adipose tissue (SC)	-	[60]
circFUT10	Up	let-7c family	PPARGGC1B	Adipocyte proliferation↑ Differentiation↓	Qinchuan cattle adipose tissue	Overexpression	[61]
circSAMD4A	Up	miR-138-5p	EZH2	Lipogenesis↑ Differentiation↑	C57BL6 mice/ obese patient VAT	Knockdown	[65]
circH19	Up	PTBP1	SREBP1	Lipogenesis↓ Differentiation↓	hADSCs/ human blood sample	Knockdown	[67,68]
ciRS-133	Up	miR-133	PRDM16	Browning↑	Male nude mice/ gastric cancer patient blood	Overexpression/ knockdown	[63]
circNrxn2 (circ005661)	Up	miR-103	-	Browning↑ M2 macrophage polarization↑	C57BL6 mice adipose tissue	Overexpression	[64]
hsa_circ_0136134	Up	-	LPL	Adipocyte hyperplasia	Human preadipocyte/ adipocyte(VAT)	-	[62]
hsa_circ_0017650	Up	-	ITIH5	Adipocyte hyperplasia	Human preadipocyte/ adipocyte(VAT)	-	[62]
circRNA9227-1	Up	hsa-mir-665	-	Adipogenesis↑	Human preadipocyte/ adipocyte(VAT)	-	[62]
circTshz2-1	Up	-	-	Adipogenesis↑	C57BL6 mice/obese patient adipose tissue (VAT and SC)	Knockdown	[66]
circArhgap5-2	Up	-	-	Adipogenesis↑	C57BL6 mice/ obese patient VAT	Knockdown	[66]

Abbreviations: ABHD5, the synthetic ligand containing protein 5 of α-β-hydrolase domain; EZH2, enhancer of zeste homolog 2; hADSCs, human adipose-derived stem cells; ITIH5, inter-alpha-trypsin inhibitor heavy chain; LPL, lipoprotein lipase; PPARGGC1B, peroxisome proliferator-activated receptor γ coactivator 1-β; PRDM16, PR domain containing 16; SCD, stearoyl-coA desaturase; SREBP1, sterol-regulatory element-binding protein 1; SC, subcutaneous; VAT visceral adipose tissue; ↑, promotion; ↓, inhibition; -, not known/not performed.

### 4.2. circRNAs in Obesity

In the past few years, as people have realized the importance of circRNAs in regulating gene expressions, they began to explore their roles in obesity. Liu et al. analyzed differential expressions of circRNAs in adipose tissues of obese and lean people and identified circSAMD4A as the most significant circRNA expressed by visceral adipose tissues in obese subjects [67]. Analysis of clinical data showed that the increase in circSAMD4A expression was significantly positively correlated with a high body mass index (BMI) and non-remission events after bariatric surgery [67]. Further, in vitro, circSAMD4A promoted adipogenesis through the miR-138-5p/EZH2 pathway [67]. Finally, AAV9-mediated downregulation of circSAMD4A corrected HFD-induced obesity, reduced food intake, increased energy expenditure, and increased insulin sensitivity in mice [67]. These results indicate that circSAMD4A could be used as a potential target for obesity treatment and a potential prognostic marker for obese patients after bariatric surgery.

Arcinas et al. also performed deep-sequencing of human and mouse visceral and subcutaneous fat to explore novel adipose circRNAs [68]. They noted that most circRNAs are downregulated in diet-induced obesity and suggested that inflammation may post-transcriptionally regulate circRNA biogenesis [68]. In contrast, circTshz2-1 and circArhgap5-2 were highly upregulated during adipocyte differentiation [68]. Either circTshz2-1 or circArhgap5-2 knockdown could inhibit adipogenesis in vivo and in vitro [68]. CircArhgap5-2 directs the global adipocyte gene program in mice and is also conserved in human adipocytes [68]. However, neither by sponging miRNAs nor encoding peptides, the regulation of circArhgap5-2 in adipogenesis remains unclear [68].

Zhu et al. found that hsa_circH19 is upregulated in the peripheral blood of patients with MetS [69]. Further silencing of circH19 promoted human adipose-derived stem cell differentiation and lipid accumulation via targeting of PTBP1 (polypyrimidine tract-binding protein 1) [69]. Without circH19, PTBP1 facilitates cleavage of the SREBP1 (sterol-regulatory element-binding proteins) precursor and assists the translocation of nSREBP1 to nuclei [70]. 

## 5. circRNAs in Hepatocyte Lipid Metabolism

### 5.1. Mechanisms of circRNAs in Hepatocellular Lipid Metabolism

Emerging studies have demonstrated that circRNAs regulate lipid metabolism via circRNA–miRNA–mRNA networks in hepatocytes (Table 2). circRNA_0046366 and circRNA_0046367 might directly sponge miR-34a to promote fatty acid β-oxidation in HepG2 cells [71,72]. Both circRNAs abolished the suppressive effect of miR-34a against peroxisome proliferator-activated receptor α (PPARα). PPARα restoration led to the transcriptional activation of genes associated with lipid metabolism, thus dramatically reducing the triglyceride content and ameliorating hepatic steatosis.

Additionally, from the results of computational data mining, circRNA_021412 underwent significant downregulation after high-fat stimulation attenuated its competitive inhibition of miR-1972 in HepG2 cells [73]. Thus, reactivated miR-1972 downregulated the lipin 1 (LPIN1) level, and this was followed by induction of the expressions of steatosis-related genes via PPARα activation [73].

Recently Chen et al. reported that silencing circRNA_0000660 remarkably affected lipid accumulation and insulin-like growth factor-binding protein-1 (Igfbp1) in AM12 hepatocytes [74]. miR-693 is a target of circRNA_0000660, as miR-693 inhibition combined with circRNA_0000660 knockdown reduced lipid accumulation and upregulated Igfbp1 levels [74]. Results revealed the novel circRNA_0000660/miR-693/Igfbp1 pathway of lipid metabolism in the liver.

### 5.2. circRNAs in Nonalcoholic Fatty Liver Disease (NAFLD)

NAFLD refers to the accumulation of excess fat in liver cells that is not caused by alcohol consumption. The more-severe form of NAFLD is called non-alcoholic steatohepatitis (NASH), which may eventually lead to complications such as cirrhosis, liver cancer, and liver failure [75]. Li et al. explored the roles and mechanisms of circRNAs in NAFLD through circRNA microarrays in an HFD mouse model [76]. CircScd1 expression was significantly lower in NAFLD tissues than in control tissues [76]. While overexpression of circScd1 inhibited lipid droplet accumulation, knockdown of circScd1 promoted hepatosteatosis and reduced expression levels of Janus kinase 2 (JAK2) and signal transducer and activator of transcription 5 (STAT5) [76]. These findings suggested novel links between circScd1 and the JAK2/STAT5 pathway and cicScd1 as potential therapeutic targets for preventing NAFLD progression.

Jin et al. profiled liver circRNA and mRNA in a NASH mouse model by a microarray and proposed the positive regulation of circRNA_002581 on cytoplasmic polyadenylation element-binding protein 1 (CPEB1) through sponging miR-122 by bioinformatics predictions [77]. Further silencing of circRNA_002581 significantly attenuated lipid droplet accumulation, eliminated liver damage, as evidenced by decreased levels of alanine aminotransferase (ALT), aspartate aminotransferase (AST), proinflammatory cytokines, apoptosis, and H_2_O_2_, and increased ATP levels in both mouse and cellular models of NASH [78]. circRNA_002581-knockdown markedly rescued impaired autophagy in both NASH mouse and cell models, manifested by increased autophagosome numbers, upregulated LC3-II/I levels, and decreased p62 levels [78]. Finally, CPEB1/PTEN/AMPK/mTOR signaling was shown to link autophagy, and circRNA_002581-knockdown restored this pathway both in vivo and in vitro [78]. Altogether, these data indicated that the circRNA_002581–miR-122–CPEB1 axis has therapeutic potential in NASH via autophagy restoration. 

**Table 2:** Circular (circ)RNAs in hepatocellular lipid metabolism.

circRNA	Expression Pattern in Disease	miRNA/ Protein	Effector Factor	Biological Function	Cell/Tissue Type	Molecular Assay	References
circRNA_0046366	Down	miR-34a	PPARα/SLC27A/CPT1A	β-oxidation↑ TG degradation↑	HepG2 hepatocytes	Overexpression	[69]
circRNA_0046367	Down	miR-34a	PPARα/CPT2/ACBD3	β-oxidation↑	HepG2 hepatocytes	Overexpression	[70]
circRNA_021412	Down	miR-1972	LPIN1	TG synthesis↓ β-oxidation↑	HepG2 hepatocytes	Overexpression	[71]
circRNA_0000660	Up	miR-693	IGFBP-1	Lipogenesis↓	AML-12 hepatocytes	Knockdown	[72]
circScd1	Down	JAK2/STAT5	-	Lipid uptake↓	AML-12 hepatocytes	Overexpression/ knockdown	[74]
circRNA_002581	Up	miR-122	CPEB1	Autophagy↓	AML-12 hepatocytes, NCTC-1469 cell/ BALB/c mice	Knockdown	[75,76]
circRNA SCAR	Down	ATP5B	mPTP	Mitochondrial ROS Fibroblast activation↓	Human and mouse primary liver fibroblasts	Overexpression/ knockdown	[78]

Abbreviations: ACBD3, acyl-CoA binding domain containing 3; CPEB1, cytoplasmic polyadenylation element-binding protein 1; CPT2, carnitine palmitoyltransferase 2; IGFBP-1, insulin-like growth factor-binding protein-1; JAK2, Janus kinase 2; LPIN1, lipin 1; mPTP, mitochondrial permeability transition pore;SLC27A, solute-carrier family 27A; STAT5, signal transducer and activator of transcription 5; PPARα, peroxisome proliferator-activated receptor-α; TG, triglyceride; ↑, increase/promotion; ↓, decrease/inhibition; -, non-observed result.

Notably, Zhao et al. recently compared circRNA expression profiles between primary fibroblasts from cirrhotic NASH patients and control subjects. Surprisingly, circRNAs encoded by the mitochondrial genome comprised a significant fraction of nearly 40% of downregulated circRNAs in NASH fibroblasts, despite being <0.1% of the overall circRNomics [79]. One of these mito-circRNAs, circRNA SCAR, was upregulated by PPAR-gamma coactivator 1α (PGC-1α), which is particularly located in mitochondria [80]. In a resting state, circRNA SCAR directly bound to ATP5B and shut down mitochondrial permeability transition pores (mPTPs) by blocking CypD–mPTP interactions in mitochondria [80]. However, lipid-induced ER stress reduced PGC-1α-mediated circRNA SCAR expression by the C/EBP homologous protein (CHOP) and consequently increased the mitochondrial ROS output [80]. ROS caused proinflammatory activation of liver fibroblasts, which led to NASH progression [80]. In an HFD mouse model, the administration of mito-nanoparticles containing circRNA SCAR overexpression vectors alleviated liver cirrhosis and IR [80]. Finally, circRNA SCAR downregulation in liver fibroblasts was closely associated with patients’ steatosis-to-NASH progression and IR [80]. Therefore, these data identify a mitochondrial circRNA, circRNA SCAR, which drives metaflammation and provides an attractive therapeutic strategy for NASH.

## 6. Conclusions

In recent years, circRNA biology and its role in metabolic diseases have been a hotbed of research. People found that circRNAs are not just rare weirdness or errors in pre-mRNA splicing but strictly regulated transcripts. They perform crucial biological functions, especially maintaining metabolic homeostasis. Although thousands of circRNAs have been identified, the field is still in its infancy. Many molecular details are still poorly understood and critical questions remain unanswered. For example, what are the integral factors that control expressions of circRNAs? How are circRNAs degraded in normal, unstressed cells? What are the factors that control the localization of circRNAs? What is the difference in selective splicing between circRNAs and linear RNAs? Of note, as most circRNAs remain in low abundance, how can many of them effectively sponge miRNAs or act as scaffolds or competitors for protein binding? In contrast, while circRNAs accumulate during aging [81,82,83], are these changes beneficial, harmful, or irrelevant? Although these questions are related to research on the basic cell physiology of circRNAs, investigating differences between diseased and normal tissues would allow us to build up a more precise and comprehensive picture, whether they are involved in cancer or metabolic diseases.

Obviously, only a minimal subset of circRNAs identified by RNA-Seq experiments have been studied so far. For this reason, in addition to circRNA being a relatively new research field, large-scale profiling and subsequent computational predictions have selected circRNA–miRNA–mRNA pathways, and these have so far not been confirmed by in vitro or in vivo experiments. Interactions of circRNAs with intricate and dynamic signaling involving mRNAs, miRNAs, and other transcription factors make it challenging to investigate their functions in metabolic disorders.

Fortunately, technological developments in the overexpression and knockdown/out of specific circRNAs have begun to succeed. People can generate specific circRNAs by self-splicing intron [84] or splint ligation [85] approaches in vitro and then subsequently add these to cells. Conversely, one can use short hairpin (sh)RNA/small interfering (si)RNA targeting back-splicing junctions or CRISPR/Cas9 genome editing [27] to remove specific circRNAs. These new tools extend the entire research direction from association studies to mechanistic exploration.

In particular, many authors claim that circRNAs can be used as biomarkers for diabetes and cardiovascular diseases. However, correlations do not imply causation. Until further experiments and comprehensive clinical verification produce clear evidence of the biological effects of circRNAs, findings from computational analyses should be treated as preliminary data and interpreted with caution. This does not mean that this type of observational clinical research is not important, but that this is merely the starting point. With additional new technologies in the next few years, our understanding of the dynamic networks of circRNAs will gradually increase, and they may be found to have novel biological roles. This will enable circRNA-based treatments to have a firmer foundation and promote their transition from “bench to bedside” to achieve precision medicine for metabolic diseases.

## Data Availability

Not applicable.
